# Supplementary figures and images for: Antibody conjugates for targeted delivery of Toll-like receptor 9 agonist to the tumor tissue
Source: PLoS One. 2023 Mar 13;18(3):e0282831. doi: 10.1371/journal.pone.0282831 (PMC10010539; doi:10.1371/journal.pone.0282831)

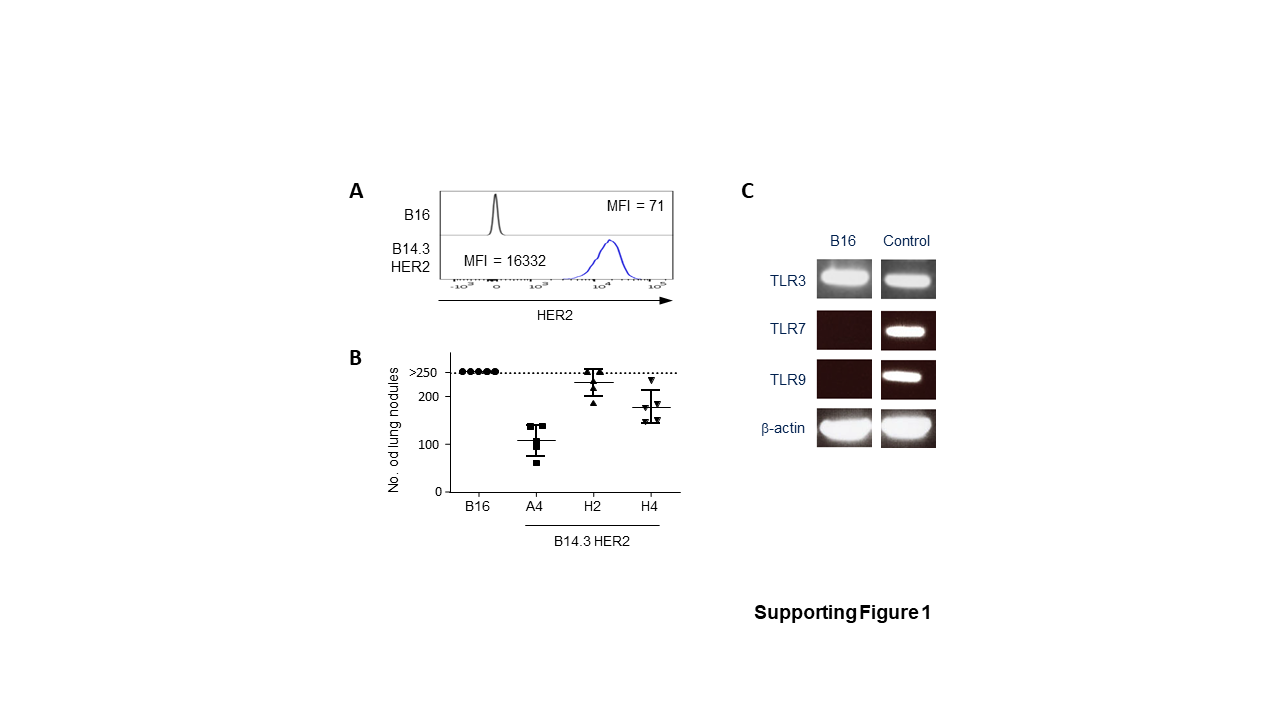

Supplement: S1 Fig — (A) Human HER2 expression of cultured B16 and B14.3 HER2 tumor cells was determined by flow cytometric analysis. (B) Lungs were harvested at day 15 post tumor inoculation. Upon fixation, the number of tumor nodules was counted, up to a maximum number of 250 nodules per mouse. (C) The expression of TLR3, TLR7 and TLR9 in B16 cells was analysed by PCR as describe previously by Edwards et al., 2003. TLR3, TLR7 and TLR9 encoding plasmids were used as control templates. Edwards AD, Diebold SS, Slack EM, Tomizawa H, Hemmi H, Kaisho T, et al. Toll-like receptor expression in murine DC subsets: lack of TLR7 expression by CD8 alpha+ DC correlates with unresponsiveness to imidazoquinolines. Eur J Immunol. 2003;33(4):827–33. (TIF) [file pone.0282831.s001.tif]

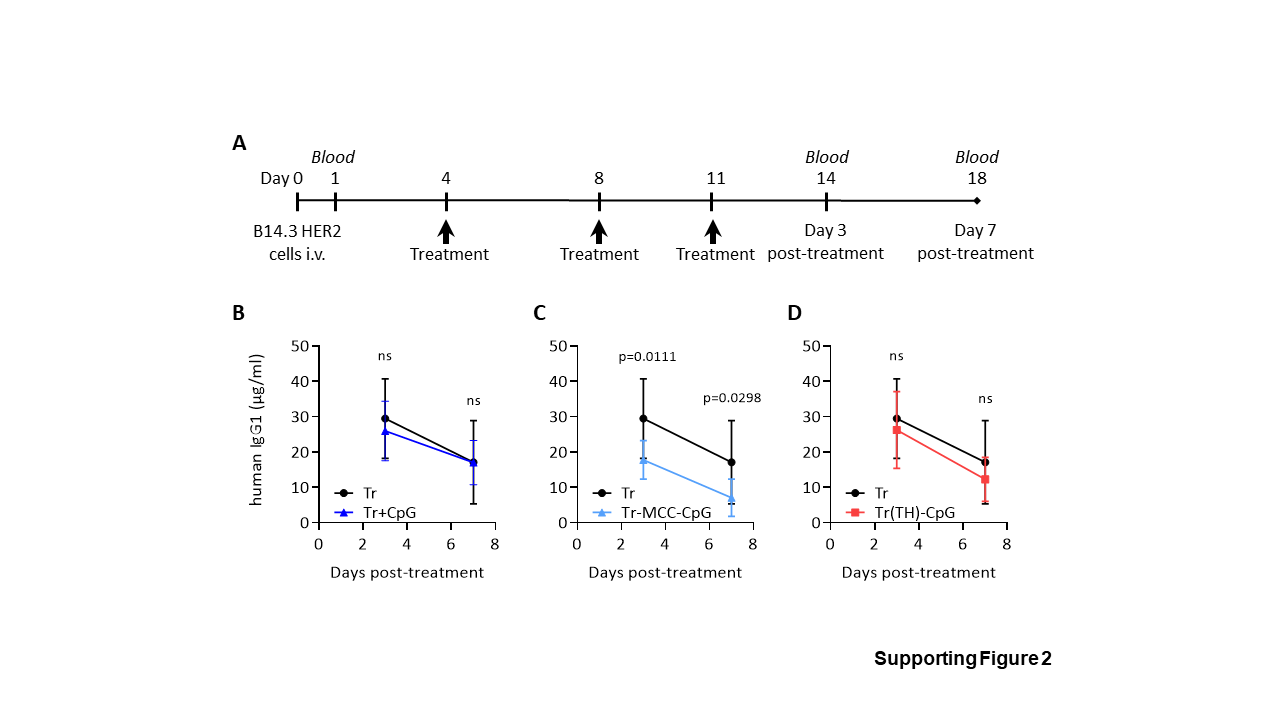

Supplement: S2 Fig — (A) Schematic overview of the experiment. Mice bearing B14.3 HER2 tumors (n = 10) were treated 3 times with 40μg Trastuzumab with or without free CpG ODN (Tr +CpG or Tr, respectively), Trastuzumab-MCC-CpG (Tr-MCC-CpG), or TH-Trastuzumab-MCC-CpG (Tr(TH)-CpG). Serum samples were harvested at days 3 and 7 after the last round of treatment. (B) The serum concentration of human IgG1 was determined by sandwich ELISA using an antibody or respective conjugate standard curve. Data are shown as mean ± SD and are pooled from 3 independent experiments. (TIF) [file pone.0282831.s002.tif]

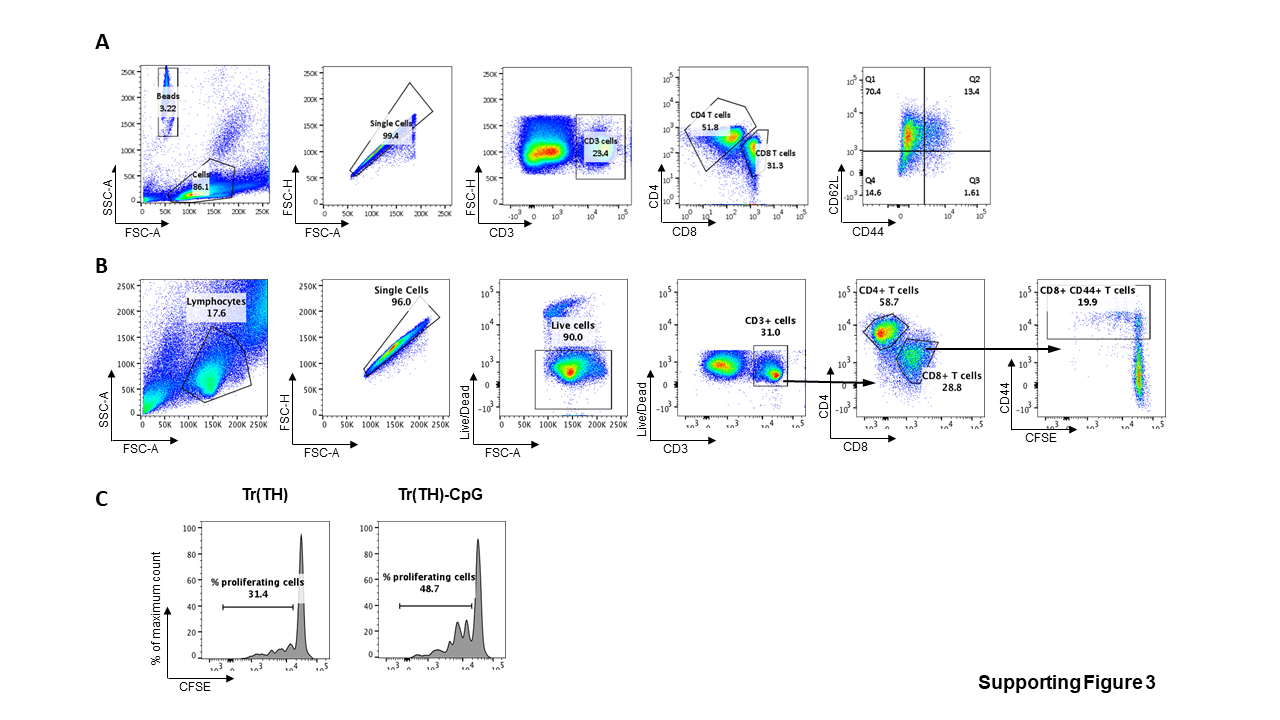

Supplement: S3 Fig — (A) For analysis of different T cell populations, splenocytes from treated mice were harvested at day 18, stained LIVE/DEAD fixable dye and CD3-, CD4-, CD8-, CD44- and CD62L-specific antibodies and analyzed by flow cytometry. The gating strategy for identifying the percentage of CD44+ CD62L+ memory T cells for CD4+ and CD8+ T cell populations is shown. FMO controls were used for gating. (B) For antigen-specific proliferation assays, splenocytes were labelled with CFSE and cultured for 3 days with GM-CSF derived BMDC loaded with tumor specific proteins and peptides. Cells were harvested and stained for CD3, CD4, CD8, CD44 and analyzed by flow cytometry. FMO controls were used for gating. (C) The percentage of CFSE diluting cells was calculated and served as a marker of proliferation. Representative histograms of activated CD8+ T cells from mice treated with TH-Trastuzumab (Tr(TH)) and TH-Trastuzumab-MCC-CpG conjugate (Tr(TH)-CpG) are shown as examples. (TIF) [file pone.0282831.s003.tif]

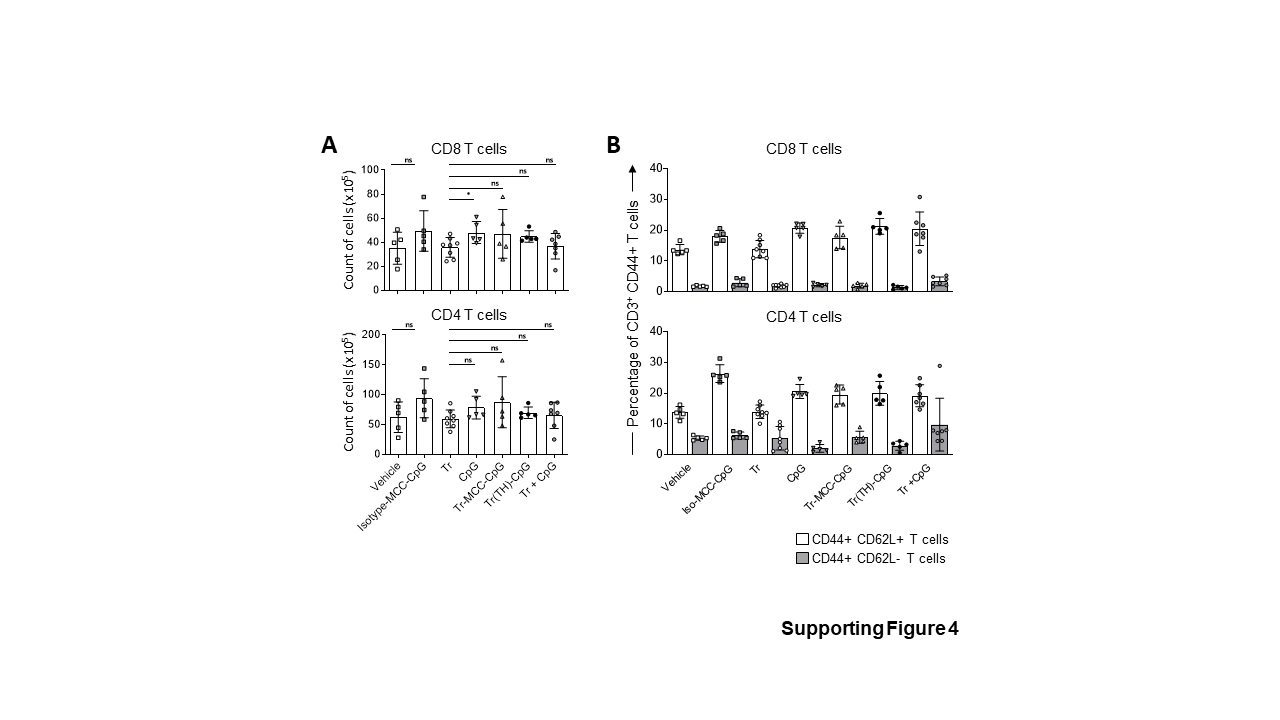

Supplement: S4 Fig — Mice were treated on days 4, 8 and 11 post tumor inoculation with 2mg/kg body weight of the indicated antibodies or antibody conjugates or with a dose of free CpG equivalent to the CpG dose applied in conjugated form. Splenocytes were isolated on day 18. Splenocytes were stained and analyzed ex vivo by flow cytometry to determine the absolute counts of (A) CD4+ versus CD8+ CD3+ T cells or (B) the percentage of CD62L+ versus CD62L− CD44+ cells within the CD4+ and CD8+ T cell populations. Each symbol represents a mouse treated with vehicle (n = 5), Iso-MCC-CpG, Tr-MCC-CpG, Tr(TH)-CpG or Tr (n = 8 for each), and Tr + CpG (n = 7). Means ± SD of samples from two independent experiments are depicted. Data were analyzed by Mann-Whitney two tailed test; *p<0.05; **p<0.01; ns—not significant. (TIF) [file pone.0282831.s004.tif]

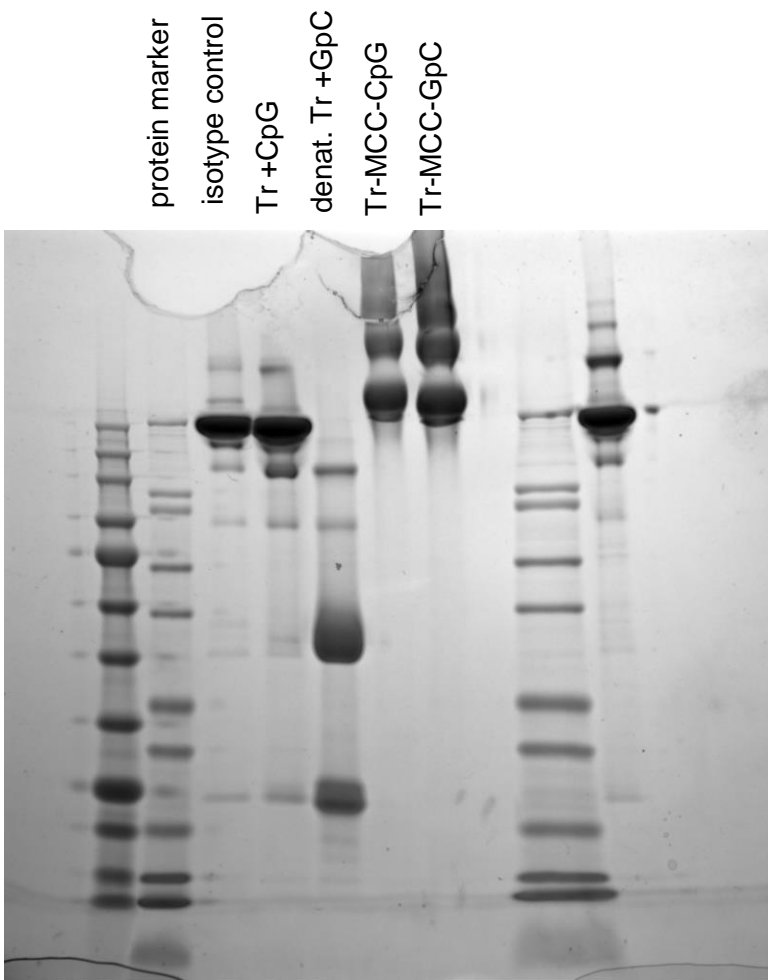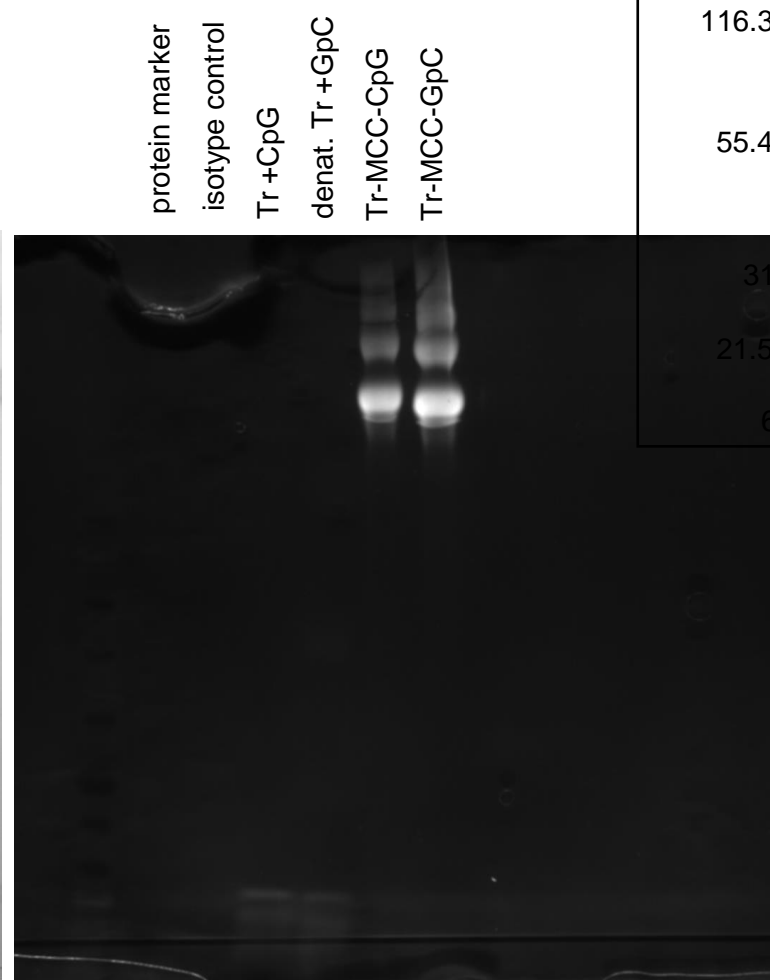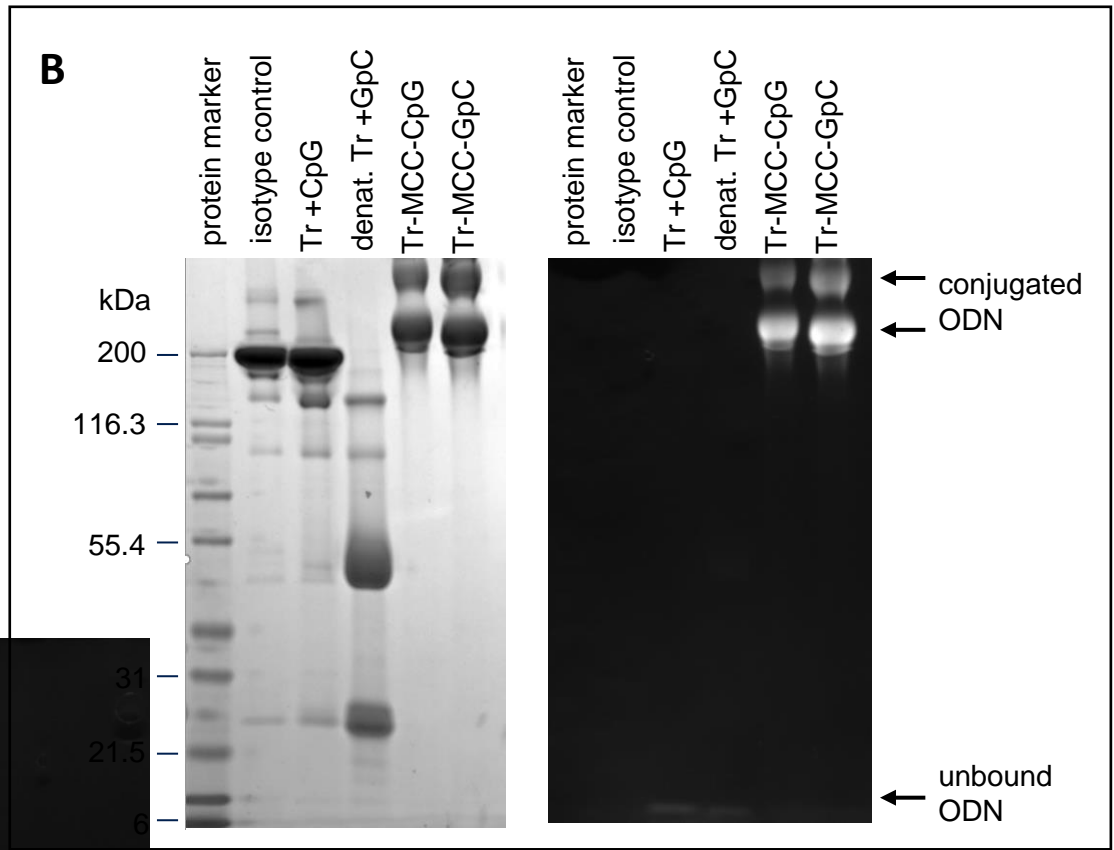

Figure 1B - Gel Plots

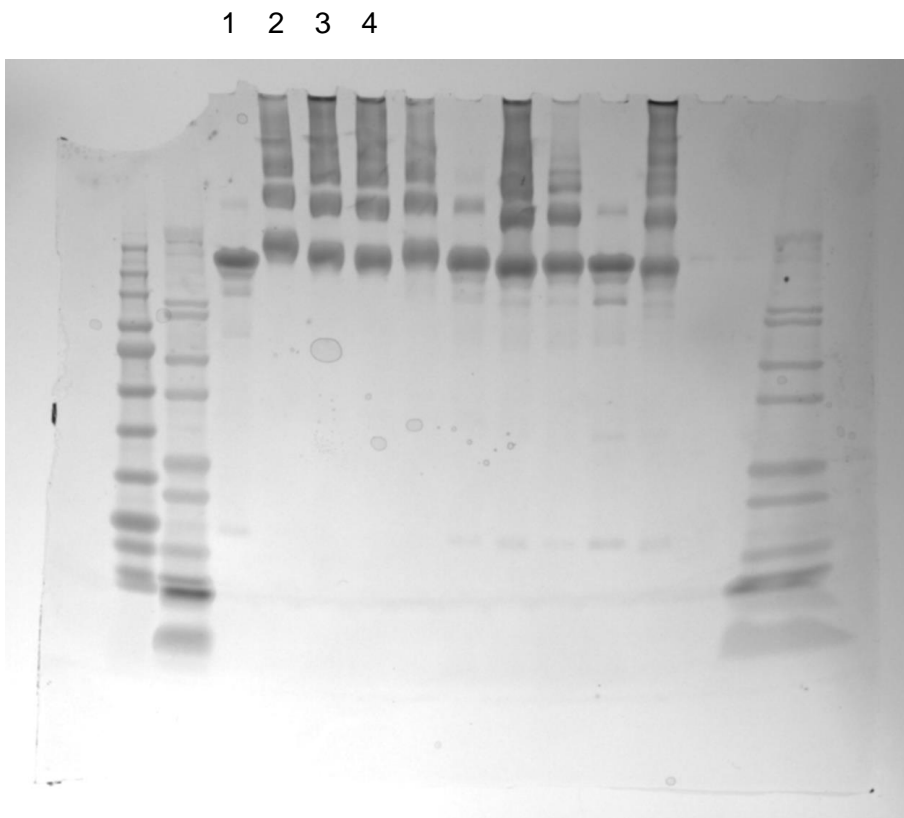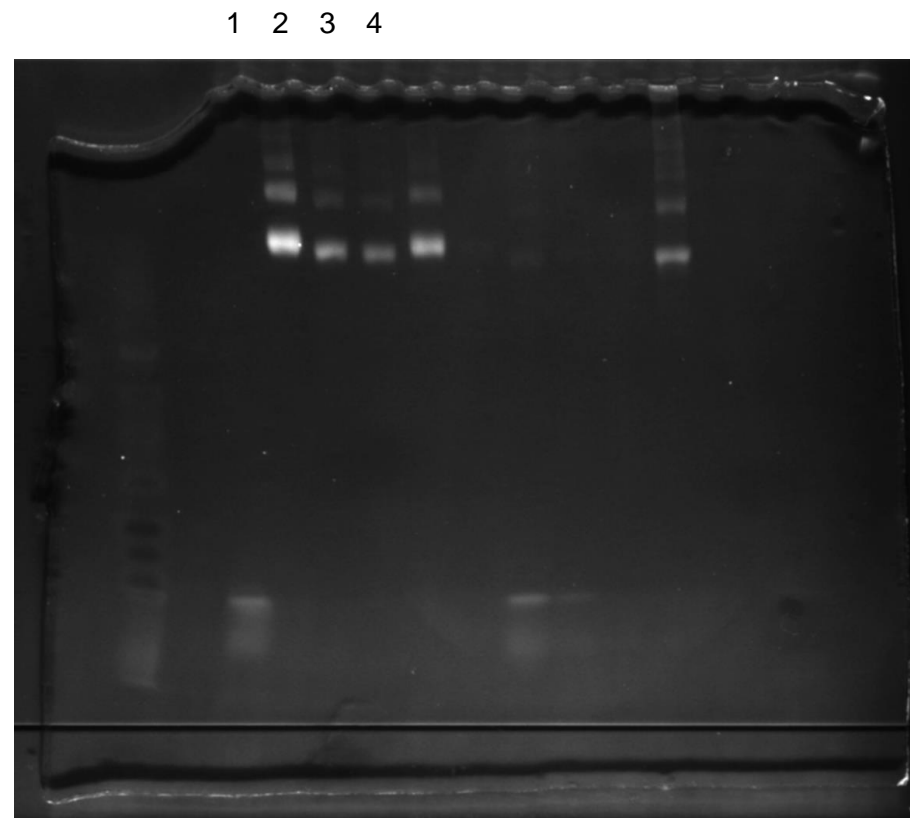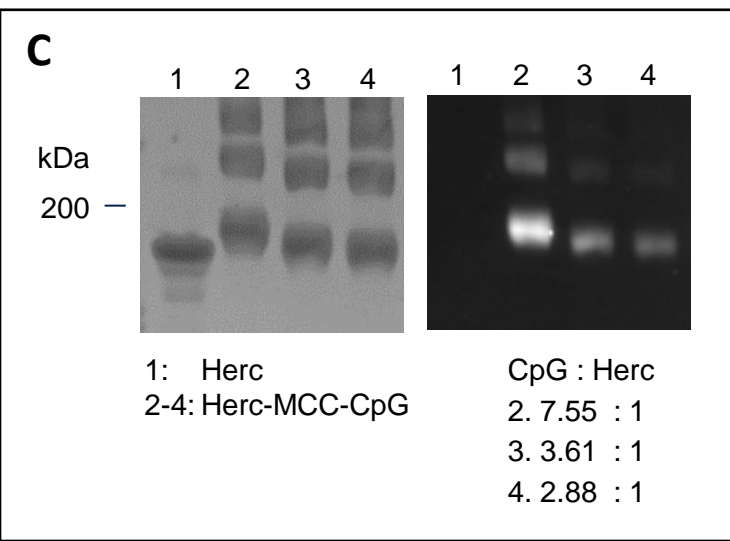

Figure 1C - Gel Plots

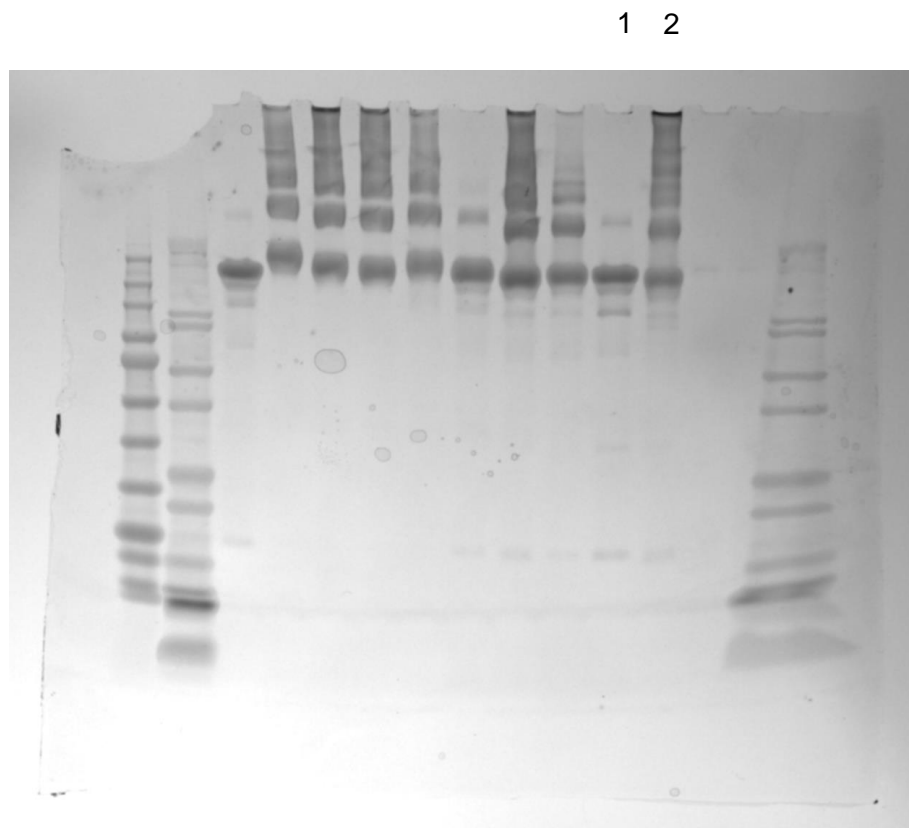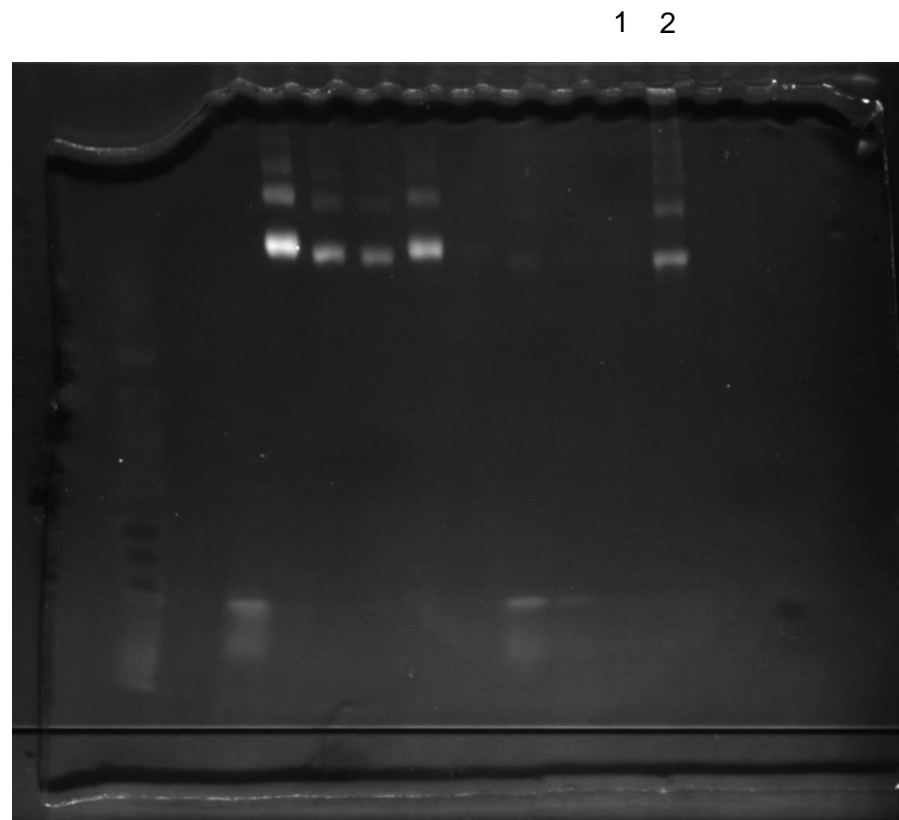

**D**

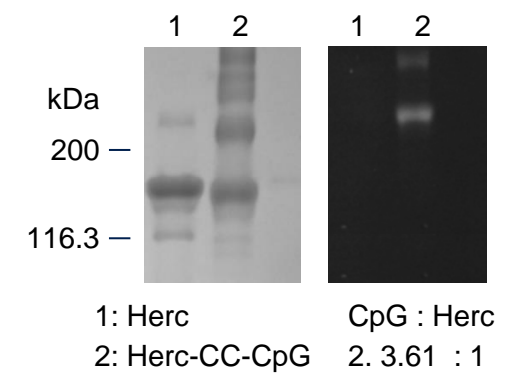

**Figure 1D - Gel Plots**

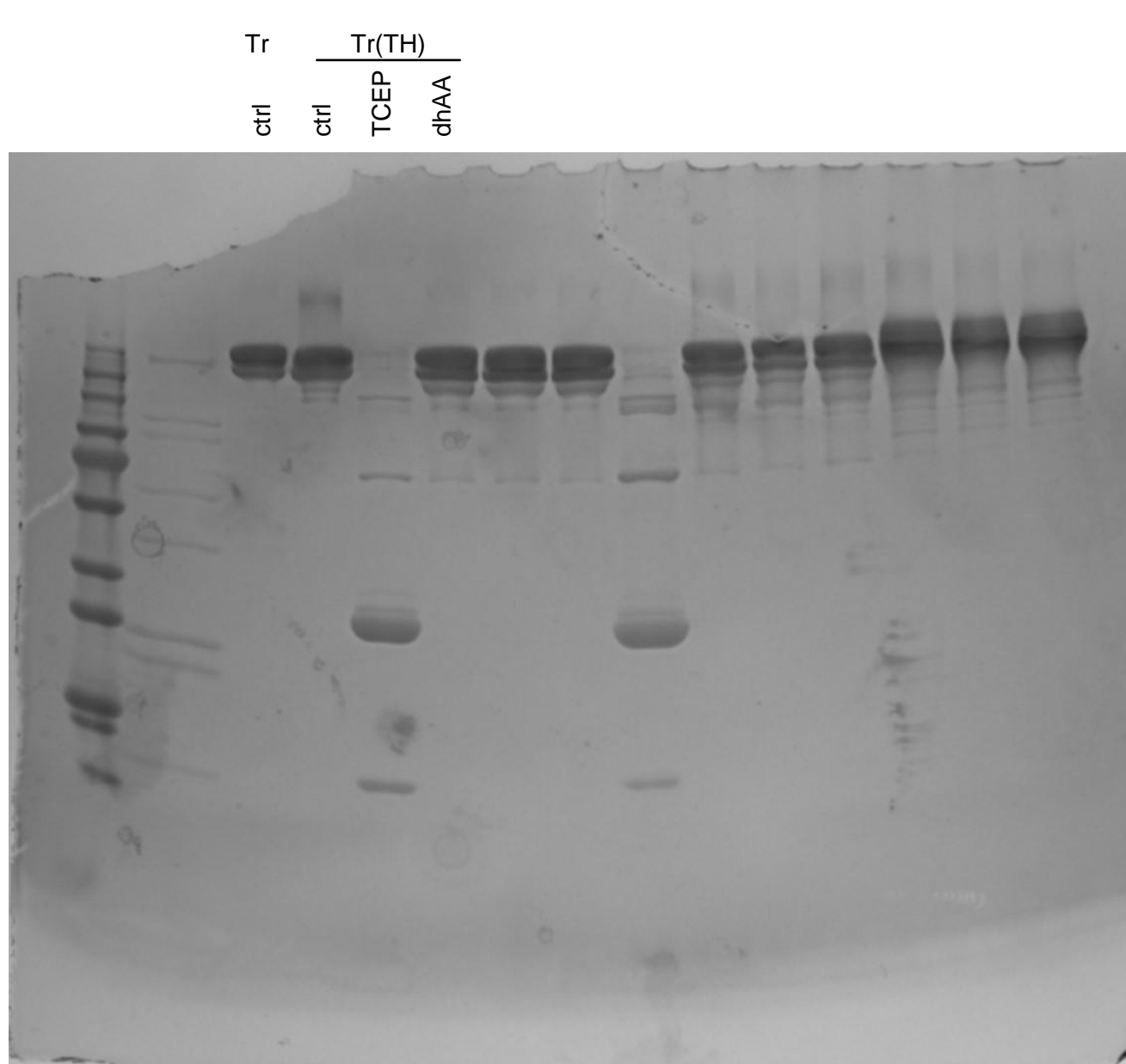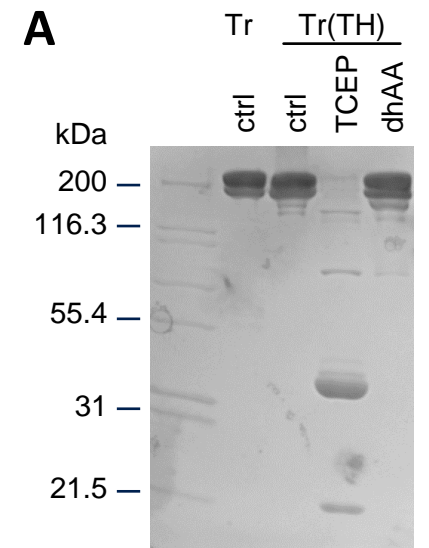

**Figure 5A - Gel Plots**

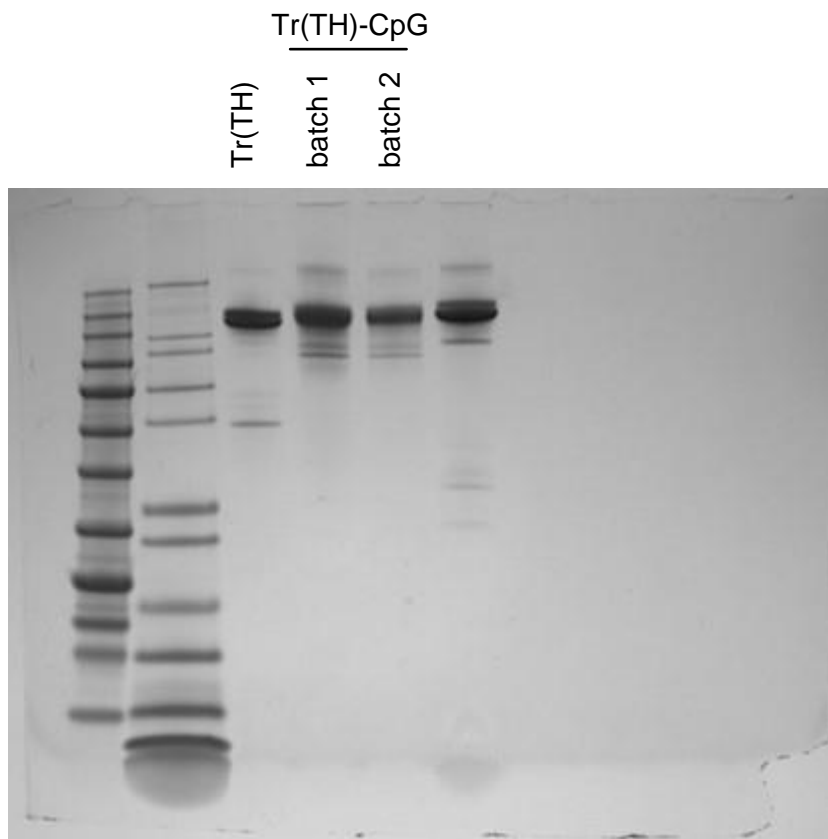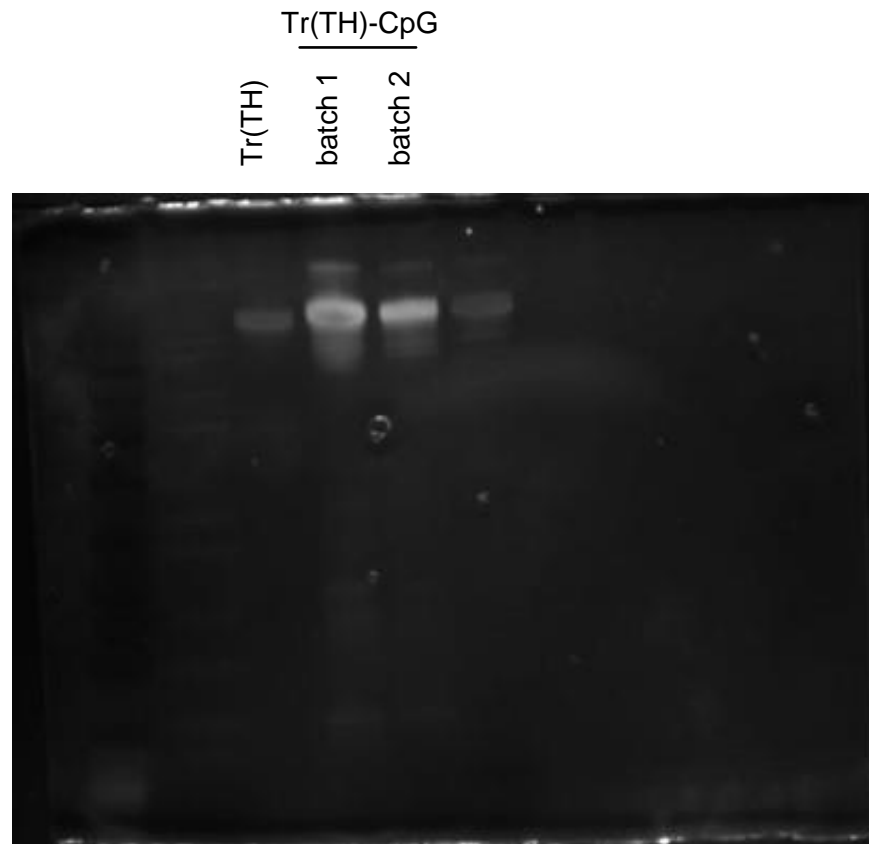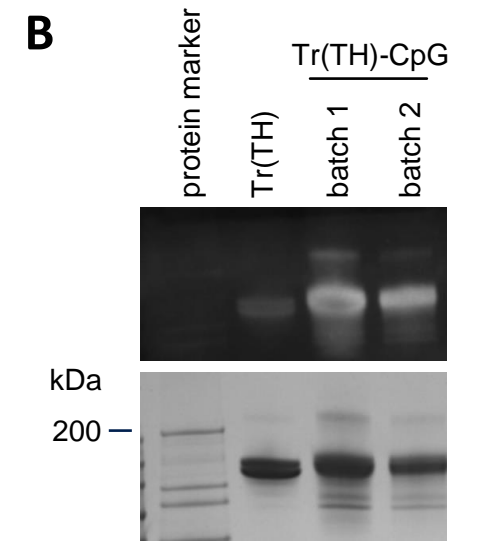

Figure 5B - Gel Plots

Supplement: S1 Raw images — (PDF) [file pone.0282831.s006.pdf]
