# Supplementary material for: Antibody conjugates for targeted delivery of Toll-like receptor 9 agonist to the tumor tissue
Source: PLoS One. 2023 Mar 13;18(3):e0282831. doi: 10.1371/journal.pone.0282831 (PMC10010539; doi:10.1371/journal.pone.0282831)
Supplement: S1 Table — Trastuzumab (Tr) and isotype control antibody (Iso) were conjugated to an excess of ODN and employing excess cross-linker SMCC at different ratios of CpG to antibody and SMCC to antibody as listed. The conjugation was performed in PBS or borate buffer as shown and the molar ratio of ODN to antibody was determined for each conjugate individually. (DOCX) [file pone.0282831.s005.docx]

| **antibody** | **conjugation buffer** | **molar excess of SMCC** | **molar excess of CpG 1668** | **molar ratio of CpG to antibody in conjugate** |
| --- | --- | --- | --- | --- |
| Tr | PBS | 34 | 10 | 7.55 |
| Tr | PBS | 34 | 6 | 3.61 |
| Tr | PBS | 34 | 3 | 2.88 |
| Tr | PBS | 10 | 3 | 0.97 |
| Tr | PBS | 34 | 10 | 9.16 |
| Tr | PBS | 34 | 6 | 6.12 |
| Tr | PBS | 34 | 3 | 3.09 |
| Iso | PBS | 34 | 6 | 2.87 |
| Tr | Borate buffer | 30 | 5 | 2.72 |
| Tr | Borate buffer | 20 | 5 | 2.13 |
| Tr | Borate buffer | 20 | 5 | 1.86 |
| Tr | Borate buffer | 10 | 5 | 1.08 |
| Tr | Borate buffer | 30 | 4 | 2.02 |
| Tr | Borate buffer | 20 | 5 | 2.12 |
| Tr | Borate buffer | 10 | 4 | 0.81 |
| Tr | Borate buffer | 5 | 4 | 0.40 |
| Iso | Borate buffer | 20 | 5 | 1.82 |
