# Supplementary material for: Antibody conjugates for targeted delivery of Toll-like receptor 9 agonist to the tumor tissue
Source: PLoS One. 2023 Mar 13;18(3):e0282831. doi: 10.1371/journal.pone.0282831 (PMC10010539; doi:10.1371/journal.pone.0282831)
Supplement: Attachment — Submitted filename: Response_To_Reviewers_230216.docx [file pone.0282831.s009.docx]

Paulo Lee Ho, Ph.D.

Academic Editor PLOS ONE

Cancer - Immunotherapy & Tumour Immunology

22 February 2023

**Subject:	Revision of manuscript PONE-D-22-14973**

Dear Dr Ho,

We would like to thank you for giving us further opportunity to submit an improved version of our manuscript entitled “Antibody conjugates for targeted delivery of Toll-like receptor 9 agonist to the tumor tissue”.

We appreciate the additional comments from reviewer 2. In response, we provide a figure and a short description of the flow cytometry assay we explored for assessment of HER2 binding by the antibody conjugates. In addition, we have revised the manuscript further to reduce the length of manuscript. As originally suggested by reviewer 1, we focused on the description and discussion of the in vivo tumor model for the shortening of the manuscript, but we also included minor revisions in other parts of the paper.

We hope that the additionally revised version of our manuscript is acceptable for publication in PLOS ONE and are looking forward to hearing from you.

Yours sincerely,

Sandra Diebold

Principal Scientist, Immunotherapy, Biotherapeutics

T	+44 (0)1707 641278

E	[sandra.diebold@nibsc.org](mailto:sandra.diebold@nibsc.org)

Point-by-point response to the additional comments from reviewer 2:

*6. Review Comments to the Author*

*Reviewer #1: (No Response)*

*Reviewer #2: The authors have made some effort to improve and shorten the manuscript.*

*Remaining issues:*

*3.2. HER2 Binding Assay
“We have undertaken flow cytometric analysis of mAb binding to cells, but have not included this in the manuscript, since it did not provide more insight into HER2 binding”.*

*Response: The authors do not have to show the data, but they should mention the outcome of the experiment at least in the rebuttal. Were the FACS stainings identical?*

Authors’ Response: Please see attachment for a response to this reviewer comment. We include a representative figure of the FACS staining, and also a brief explanation of the results. The flow cytometry assay supports the conclusions drawn from the HER2 binding assay, however, we had concerns that minor differences in binding of different stochastic conjugates that we observed in the HER2 binding assay were not reliably quantifiable in the flow cytometry assay.

Please Note: The attachment is included in the submission for review by the reviewers only and is not meant to be included as supplementary information as part of the manuscript.

*3.6. Length of Manuscript
In order to shorten the manuscript, we removed a sentence on page 21 which we felt did not add to a better understanding of the study. However, we felt unable to identify further superfluous text passages. Moreover, both reviewers requested further clarifications and discussion points to be included in the manuscript.

Response: This is really a minor shortening. The last reviewer comment concerning the murine models and immunomonitoring (see below) was not answered by the authors and included some suggestions to shorten.

The murine models are quite artificial both from tumor (no direct effect on B16) as the immune component and results appear quite variable within groups Could the authors comment on the variation in the groups? In my opinion the addition of all the immunomonitoring in this model is a bit difficult to support as the model is so artificial. Maybe better to shorten and focus on the conjugate characterization parts.*

Authors’ Response: Yes, reviewer 1 was correct in pointing out that the groups are very variable. The high variability was seen in all experiments and in all treatment groups and the reason for this high variability is currently unknown. Injections were performed by highly trained and experienced animal technicians, and we do not suspect poor injection technique to play a role in the high variability observed. One possible explanation is that the human HER2-expressing modified melanoma cells are inherently more immunogenic than the parental cell line and that in a fraction of mice immune responses directed against the human neoantigen develop spontaneously. We now mention this in the discussion of the revised version of the manuscript on page 32.

While the model is artificial, the results are significant despite the high variability within groups. In our discussion, we have been open about the pros and cons of this model and, also discuss the pros and cons of alternative models such as the humanised model. To our knowledge there is no ideal animal model to study the function of a therapeutic antibody such as Trastuzumab targeting a human tumour marker. Humanised mouse models are also highly artificial and have their own caveats.

In order to comply with the reviewers’ request to shorten the manuscript, we have revised the manuscript further by simplifying the introduction of the tumour model on page 24 and by editing the presentation of the results on pages 25-28. We also revised the discussion of the tumour model on pages 32-33 in order to shorten the manuscript further. Furthermore, we have made deletions in other parts of the text and have moved a paragraph from the result section to the materials & method section. We were anxious to not delete any relevant observations and discussion points regarding the tumour model from the manuscript. We hope that this level of shortening of the manuscript is acceptable and we would like to point out that PLOS ONE does not have restrictions on the length of articles.

Journal Requirements

We have checked our reference list. All references appear on PubMed except for the article by Nunes et al., 2017 (reference 29). We checked reference 29 on the Royal Society of Chemistry website where the article is freely available:

[Use of a next generation maleimide in combination with THIOMAB™ antibody technology delivers a highly stable, potent and near homogeneous THIOMAB™ antibody-drug conjugate (TDC) - RSC Advances (RSC Publishing)](https://pubs.rsc.org/en/content/articlelanding/2017/RA/C7RA04606E) .

We have therefore not revised the reference list but would be happy to do so if there are concerns about a specific reference.
